# Empagliflozin Reduces the Progression of Hepatic Fibrosis in a Mouse Model and Inhibits the Activation of Hepatic Stellate Cells via the Hippo Signalling Pathway

**DOI:** 10.3390/biomedicines10051032

**Published:** 2022-04-29

**Authors:** Yu-Jung Heo, Nami Lee, Sung-E Choi, Ja-Young Jeon, Seung-Jin Han, Dae-Jung Kim, Yup Kang, Kwan-Woo Lee, Hae-Jin Kim

**Affiliations:** 1 Department of Endocrinology and Metabolism, Ajou University School of Medicine, Suwon 16499, Korea; gotonature@ajou.ac.kr (Y.-J.H.); chamsaram6@ajou.ac.kr (N.L.); jjy@aumc.ac.kr (J.-Y.J.); hsj@ajou.ac.kr (S.-J.H.); djkim@ajou.ac.kr (D.-J.K.); lkw65@ajou.ac.kr (K.-W.L.); 2 Department of Physiology, Ajou University School of Medicine, Suwon 16499, Korea; choise@aumc.ac.kr (S.-E.C.); kangy@ajou.ac.kr (Y.K.)

**Keywords:** hepatic fibrosis, Hippo signalling pathway, hepatic stellate cells, empagliflozin

## Abstract

Hepatic fibrosis is the excessive production and deposition of the extracellular matrix, resulting in the activation of the fibrogenic phenotype of hepatic stellate cells (HSCs). The Hippo/Yes-associated protein (YAP) signalling pathway is a highly conserved kinase cascade that is critical in regulating cell proliferation, differentiation, and survival, and controls stellate cell activation. Empagliflozin, a sodium-glucose cotransporter type-2 inhibitor, is an antidiabetic drug that may prevent fibrotic progression by reducing hepatic steatosis and inflammation. However, little is known about its mechanism of action in liver fibrosis. In this study, we used male C57 BL/6 J mice fed a choline-deficient, l-amino acid-defined, high-fat diet (CDAHFD) as a model for hepatic fibrosis. For 5 weeks, the mice received either a vehicle or empagliflozin based on their assigned group. Empagliflozin attenuated CDAHFD-induced liver fibrosis. Thereafter, we identified the Hippo pathway, along with its effector, YAP, as a key pathway in the mouse liver. Hippo signalling is inactivated in the fibrotic liver, but empagliflozin treatment activated Hippo signalling and decreased YAP activity. In addition, empagliflozin downregulated the expression of pro-fibrogenic genes and activated Hippo signalling in HSCs. We identified a mechanism by which empagliflozin ameliorates liver fibrosis.

## 1. Introduction

Hepatic fibrosis, the most common stage of chronic liver disease, is a serious global problem [1]. Hepatic fibrosis is caused by excessive scarring due to the overproduction and deposition of the extracellular matrix (ECM), including fibrillar collagens, which occurs in most types of chronic inflammatory liver injuries [2]. Liver injury stimulates the recruitment of inflammatory cells and activates fibrogenic cells [3]. Hepatic stellate cells (HSCs) are mainly used to investigate hepatic fibrosis [4] because the activation of HSCs is involved in proliferation, fibrogenesis, matrix degradation, cytokine release, and α-smooth muscle actin (*α-SMA*) overexpression [1,5].

The intracellular Hippo signalling pathway regulates the growth of tissues and organs by regulating cell proliferation and death [6]. In mammals, the key enzymes in the Hippo signalling pathway are the mammalian sterile 20-related kinases 1 and 2 (MST1 and MST2) and large tumour suppressor kinases 1 and 2 (LATS1 and LATS2) [7]. The canonical Hippo pathway requires a WW-domain containing two adaptor proteins: the scaffold protein Salvador (SAV1) and Mps One Binder 1 (MOB1) [8]. In contrast to the canonical hippo pathway, the other signalling cascades that involve MST1/2 but do not regulate LAST1/2 kinase were recently identified as the alternative Hippo pathway, which includes additional kinases, such as the nuclear Dbf2-related kinases 1 and 2 (NDR1/2) [9]. In the canonical Hippo pathway, the activation of the MST1/2–LATS1/2 kinase cascade regulates the localisation of the Yes-associated protein (YAP) and transcriptional co-activator with the PDZ-binding motif (TAZ) [8,10,11,12,13,14]. When dephosphorylated, YAP/TAZ translocate into the nucleus and interact with TEAD1-4 and other transcription factors to induce the expression of genes, including amphiregulin (*AREG*), connective tissue growth factor (*CTGF*), and cysteine-rich angiogenic inducer 61 (*CYR61*) [15]. A deficiency in liver Mst1/Mst2 causes the dephosphorylation of YAP (Ser127) in the cytoplasm and increases YAP nuclear localisation [8]. Mannaerts et al. showed that YAP activation promotes the earliest changes in gene expression during HSC activation and fibrosis [16].

Sodium–glucose cotransporter 2 (SGLT2) inhibitors are antidiabetic agents that have been reported to improve non-alcoholic fatty liver disease (NAFLD) and reduce aspartate transaminase levels and hepatic steatosis in patients with NAFLD and type 2 diabetes mellitus (T2DM) [17,18]. Recently, empagliflozin (EMPA) treatment has been associated with the improvement of hepatic steatosis and fibrosis in patients with NAFLD and T2DM [19]. EMPA reduces hepatic steatosis associated with the activation of AMP-activated protein kinase (AMPK) in db/db mice [20]. Previously, we have reported that EMPA can exhibit anti-inflammatory effects via the inhibitor of NF-κB kinase/nuclear factor-κB (IKK/NF-κB), mitogen-activated protein kinase kinase 7/c-Jun N-terminal kinases (MKK7/JNK), and the Janus kinase 2/Signal transducer and activator of transcription 1 (JAK2/STAT1) signalling pathways in RAW264.7 cells [21]. In our previous studies using a choline-deficient, L-amino acid-defined, high-fat diet (CDAHFD)-induced NAFLD mouse model, EMPA ameliorated the progression of NAFLD, including fat accumulation, inflammation, and fibrosis [22]. However, there are very few studies on hepatic-fibrosis-related EMPA activity, and its molecular mechanisms have not been completely verified.

In the present study, we demonstrated that EMPA attenuated CDAHFD-induced liver fibrosis in vivo and inhibited HSC proliferation and activation in vitro. Our data suggest that the phosphorylation of the Hippo protein is downregulated in CDAHFD-induced liver fibrosis. Furthermore, EMPA inhibited the proliferation and activation of HSCs by activating the Hippo signalling pathway.

## 2. Materials and Methods

### 2.1. Animal Study

The C57 BL/6 J mice, 5-week-old males, were obtained from OrientBio Inc (Sungnam, Korea). Mice were housed in a temperature-controlled room (22 ± 2 °C) with a 12 h light/dark cycle and fed ad libitum. Following two weeks of acclimatisation, mice were assigned into the following three groups: (ⅰ) vehicle (dimethylsulfoxide)/control diet group (CD/Veh; *n* = 8), (ⅱ) vehicle/CDAHFD group (CDAHFD/Veh; *n* = 8), (ⅲ) EMPA/CDAHFD group (CDAHFD/EMPA; *n* = 8). In the CD/Veh group, mice were fed normal chow diet containing 10 kcal% fat (D12450B; Research Diets Inc., New Brunswick, NJ). In the CDAHFD group, mice were fed a diet with 60 kcal% fat (mostly from palm oil), 0.1% methionine, and no added choline (A19053002, Research Diets Inc., New Brunswick, NJ, USA). Treated groups were fed every day through an oral gavage with EMPA (Boehringer Ingelheim Pharma GmbH & Co. KG, Ingelheim, Germany) or vehicle for 5 weeks (10 µg/g per day by oral gavage) [23]. All animal care and experiments were conducted according to the Ajou Institutional Animal Care guidelines and were approved by the Ajou Institutional Animal Care Committee (Approval No. 2019-0013, 28 October 2019).

### 2.2. Cell Culture

The LX-2 human HSC line was generously donated by Professor Sang Geon Kim (College of Pharmacy, Seoul National University, Seoul, Korea). Cells were cultured in high-glucose Dulbecco’s modified Eagle’s medium (Welgene Inc., Daegu, Korea) supplemented with 10% (*v*/*v*) foetal bovine serum (Gibco, Grand Island, NY, USA) and antibiotics (10 μg/mL streptomycin and 100 IU/mL penicillin) at 37 °C and 5% CO_2_ in a 95% humidified atmosphere. Cells were subcultured three times a week and maintained at a confluence below 80–90% prior to administration of the EMPA (AdooQ Bioscience, Irvine, CA, USA).

### 2.3. Histopathological Staining

The livers of mice were excised for each group, fixed in 4% formalin, and embedded in paraffin. Sectioned tissues were stained with haematoxylin and eosin (H&E) and Sirius Red. The hepatic fibrosis grade was analysed using Sirius-Red-stained tissues. Immunohistochemical staining was performed by incubating the tissue sections with a primary antibody against αSMA (dilution 1:500, Abcam, Cambridge, UK) in a moisturised chamber at 4 °C overnight. After that, they were incubated with a horseradish peroxidase-conjugated anti-rabbit secondary antibody (Dako, Glostrup, Denmark).

### 2.4. Western Blotting

Total protein was extracted from the livers of mice in each group or cells using the RIPA buffer with a protease inhibitor cocktail (Roche Applied Science, Mannheim, Germany). Equivalent amounts of protein in sodium dodecyl sulphate (SDS) sample buffer (50 mmol/L Tris-HCl at pH 6.8, 2% SDS, 100 mmol/L DL-dithiothreitol, and 10% glycerol) were separated using SDS-polyacrylamide gel electrophoresis and transferred onto polyvinylidene difluoride membranes (Millipore, Billerica, MA, USA). Subsequently, the membranes were blocked with 5% (*w*/*v*) bovine serum albumin for 30 min, and target antigens were incubated overnight with primary antibodies, listed below, at 4 °C. The following antibodies were purchased from Cell Signaling Technology (Danvers, MA, USA): rabbit anti-YAP (Cat. no. 14074), rabbit anti-p-YAP (Ser127) (cat. no. 13008), rabbit anti-MOB1 (cat. no. 13730), rabbit anti-p-MOB1 (Thr35) (cat. no. 8699), rabbit anti-MST1 (cat. no. 3682), rabbit anti-MST2 (cat. no. 3952), anti-p-MST1/2 (Thr183/Thr180) (cat. no. 49332), rabbit anti-LATS1 (cat. no. 3477), and rabbit anti-p-LATS1 (Thr1079) (cat. no. 8654). The mouse anti-cTGF- (cat. no. Sc-365970) and goat anti-β-actin (cat. no. Sc-1615) antibodies were purchased from Santa Cruz Biotechnology (Santa Cruz, CA, USA). The following antibodies were purchased from Abcam: rabbit anti-α-SMA (cat. no. ab5694), rabbit anti-FN (cat. no. ab2413), and rabbit anti-collagen1α1 (cat. no. ab34710). Blots were incubated with secondary antibodies (horseradish peroxidase-conjugated anti-mouse IgG or anti-rabbit IgG) (Bethyl Laboratories, Montgomery, TX, USA) in 5% skim milk. The proteins were detected with ECL (Amersham Pharmacia Biotech, Piscataway, NJ, USA).

### 2.5. Quantitative Real-Time Polymerase Chain Reaction (qRT-PCR)

Total RNAs were extracted from the livers of mice in each group or cells with the RNAiso Plus reagent (TaKaRa Bio, Otsu, Japan). RNA concentrations were determined using a NanoDrop 1000 spectrophotometre (Thermo Fisher Scientific, Waltham, MA, USA). cDNA was synthesised using the avian myeloblastosis virus (AMV) reverse transcriptase (Beamsbio, Seoul, Korea) and random 9-mers (TaKaRa Bio, Otsu, Japan) and used as a template for PCR amplification. qRT-PCR was performed according to the instructions of the SYBR Green Master Mix using a TaKaRa TP-815 instrument by amplifying the primers listed in Table S1. DNA was amplified under the following conditions: denaturation at 95 °C for 5 min followed by 40 cycles of denaturation at 95 °C for 30 s, annealing at 60 °C for 30 s, and extension at 72 °C for 1 min. The comparative Ct method (ΔΔCt) was used to quantify gene expression, and the relative quantification was calculated as 2^−ΔΔCt^. GAPDH gene amplification was used as a reference standard to normalise the target signal. Amplification specificity was controlled by melting curve analysis.

### 2.6. Cell Viability Assay

LX-2 cells were seeded into 96-well plates (5000 cells/well), incubated overnight, and treated with or without EMPA. Aliquots measuring 10 μL of the cell counting kit-8 (CCK-8) reagent (Dojindo Laboratories, Kumamoto, Japan) were added to each well, and plates were incubated for 4 h at 37 °C following the manufacturer’s instructions. Optical density was measured at 450 nm using a microplate reader (iMark^™^, Bio-Rad Laboratories, Hercules, CA, USA).

### 2.7. Statistical Analysis

All data were expressed as the mean ± standard deviation (SD) from at least three independent experiments. The experimental data were analysed using the Student’s *t*-test, and statistical significance was set at *p*-value of 0.05 or less.

## 3. Results

### 3.1. Empagliflozin Attenuates Hepatic Fibrosis in CDAHFD-Induced Mice

**Figure 1:**
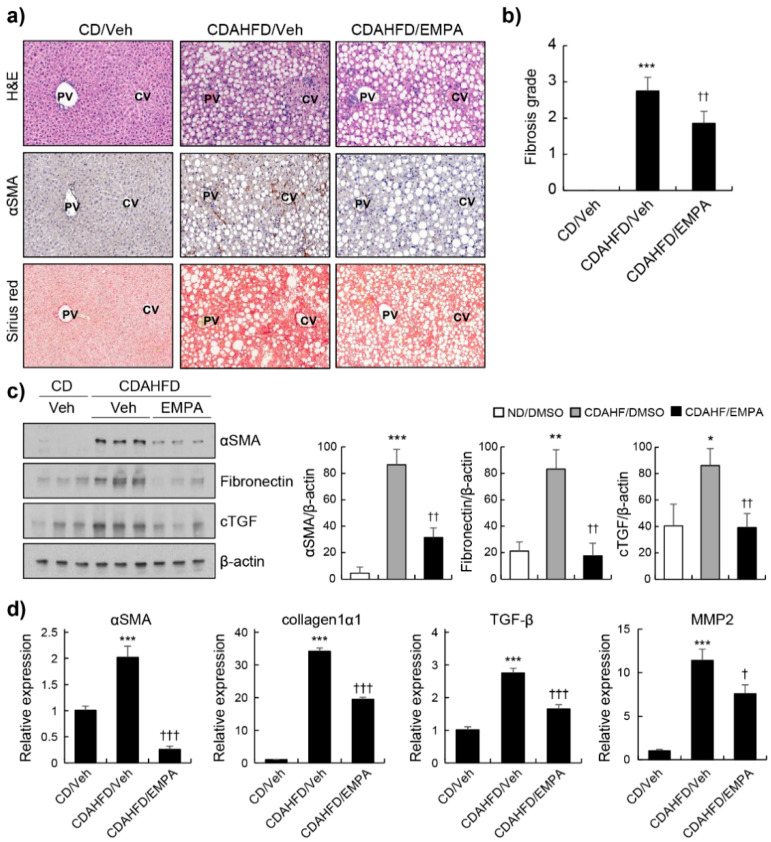
Effects of empagliflozin on hepatic fibrosis in CDAHFD-induced mice. The mice were treated with the control chow diet (CD), choline-deficient, L-amino acid-defined, high-fat diet (CDAHFD), or CDAHFD with empagliflozin (EMPA) treatment. (**a**) The liver histology was studied via macroscopic morphological examination, H&E staining, and Sirius Red staining. α-SMA signals were visualised via immunohistochemistry. (**b**) Fibrosis grades in the different groups. (**c**) α-SMA, fibronectin, and connective tissue growth factor (cTGF) signals were examined and quantified using Western blot. β-actin served as the loading control. (**d**) Relative expression of genes related to fibrosis was determined via RT-PCR. * *p* < 0.05, ** *p* < 0.01, and *** *p* < 0.001 in the comparisons of the CD-fed mice with the CDAHFD-fed mice. † *p* < 0.05, †† *p* < 0.01, and ††† *p* < 0.001 in the comparisons of the CDAHFD-fed mice with the EMPA treatment groups. PV: portal vein; CV: central vein.

A previous report demonstrated that EMPA reduced hepatic steatosis and NAFLD activity score in CDAHFD-induced NAFLD mice [22]. To investigate the effects of EMPA on the progression of liver fibrosis, mice were fed CDAHFD for 7 weeks and orally administered EMPA (10 μg/g) every day for 5 weeks [23]. First, we assessed the histopathological appearance of representative livers from mice treated with the CD/Veh, CDAHFD/Veh, or CDAHFD in combination with EMPA. H&E staining was performed to show a representative liver section morphology. Immunohistochemical staining of α-SMA, a marker of HSC activation, showed an increased expression of α-SMA in the livers of CDAHFD/Veh mice compared to that in CD/Veh mice. The EMPA-treated group showed a significantly reduced α-SMA expression (Figure 1a). Sirius Red staining detected the collagen fibre deposition in liver tissues. Fibrotic connective tissue accumulation was significantly higher in CDAHFD-fed mice than in CD-fed mice. The mean fibrosis grade of the CDAHFD-fed mice was 2.8 ± 0.37, whereas that of the EMPA-treated groups decreased to 1.9 ± 0.33 (Figure 1b). The results were further confirmed via Western blotting and RT-PCR. EMPA treatment significantly reduced the levels of α-SMA, fibronectin, and connective tissue growth factor (cTGF) in the livers of CDAHFD-fed mice (Figure 1c). Pro-fibrotic genes, such as *α-SMA*, collagen 1 α1, *TGF-β*, and matrix metallopeptidase 2 (*MMP2*), were overexpressed in CDAHFD-fed mice. EMPA treatment significantly reduced the expression of profibrotic genes (Figure 1d).

### 3.2. Empagliflozin Decreased Fibrosis Markers and Proliferation in HSCs

**Figure 2:**
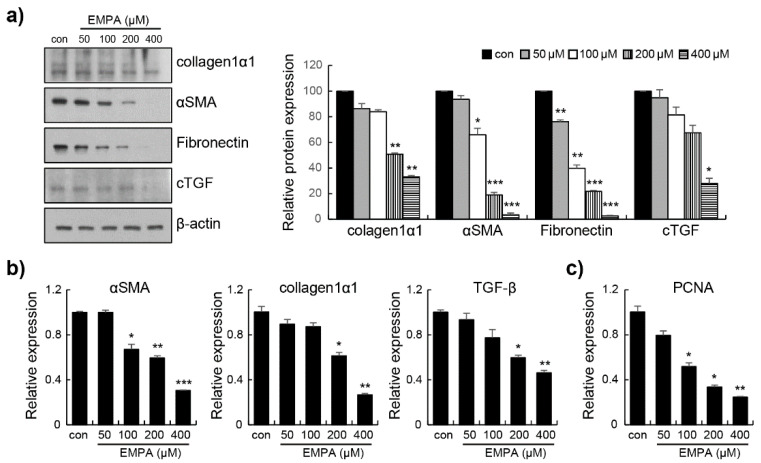
Empagliflozin downregulates pro-fibrogenic markers and decreases proliferation in LX-2 cells. (**a**) Collagen 1 α1, α-SMA, fibronectin, and cTGF were assayed by Western blotting using specific antibodies. The maximum control phosphoprotein intensity was set to 100%, and relative test intensities were calculated. (**b**) The relative expression of *α-SMA*, Collagen 1 α1, and *TGF-β* was determined via real-time polymerase chain reaction (RT-PCR). (**c**) The cell proliferation marker, proliferating cell nuclear antigen (*PCNA*), was determined via RT-PCR. Data are expressed as means ± standard deviation of three independent experiments. * *p* < 0.05, ** *p* < 0.01, and *** *p* < 0.001 compared to the control.

A key feature of hepatic fibrosis is that HSCs induce the excessive deposition of ECM proteins. The sustained activation of HSC involves changes in cell proliferation, chemotaxis, and fibrogenesis [2,3]. Western blotting revealed that the activation of collagen 1 α1, α-SMA, fibronectin, and cTGF was significantly reduced (Figure 2a). We investigated the effects of EMPA on the expression of pro-fibrogenic genes in LX-2 cells. The expression of pro-fibrotic genes, such as *α-SMA*, collagen 1 α1, and *TGF-β*, was decreased by EMPA in LX-2 cells (Figure 2b). In addition, EMPA inhibited the expression of the proliferating cell nuclear antigen (*PCNA*) in LX-2 cells in a dose-dependent manner (Figure 2c). These results indicate that the proliferation of activated HSCs was suppressed by EMPA. Toxicity analysis using CCK-8 showed no difference between cells treated with any of the EMPA concentrations compared to untreated cells (Figure S1).

### 3.3. Empagliflozin Activated the Hippo Signalling Pathway in CDAHFD-Induced Mice

**Figure 3:**
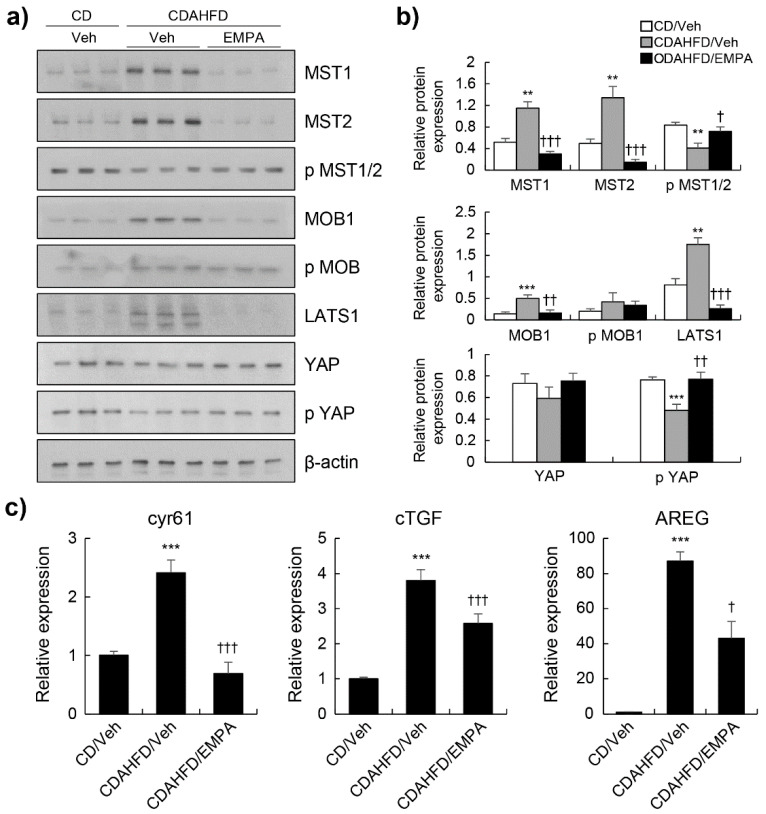
Effect of EMPA on Hippo signalling pathway in CDAHFD-induced mice. (**a**) Western blot of mice liver protein extracts with the indicated antibodies. (**b**) Quantitation and normalisation of phospho-specific signal to total protein or total protein to β-actin from a. (**c**) The relative expression of *cyr61*, *cTGF*, and *AREG* was determined via real-time PCR. ** *p* < 0.01, and *** *p* < 0.001 in the comparisons of the CD-fed mice with the CDAHFD-fed mice. † *p* < 0.05, †† *p* < 0.01, and ††† *p* < 0.001 in the comparisons of the CDAHFD-fed mice with the EMPA treatment groups.

The Hippo signalling pathway regulates cellular processes, including cell survival, proliferation, differentiation, and organ growth. The activation of the MST1/2 and LATS1/2 kinases cascade is the main initiating event in Hippo signalling; it also regulates the localisation of YAP [7,8]. We investigated whether EMPA phosphorylates YAP through the canonical Hippo pathway in CDAHFD-fed mice. CDAHFD-fed mice showed significantly enhanced levels of MST1/2, MOB1, and LATS1 and decreased levels of p-MST1/2 and p-YAP compared with those in the CD-fed mice (Figure 3a,b). As shown in Figure 3a,b, the levels of MST1/2, MOB1, and LATS1 were markedly reduced by EMPA treatment compared to those of the CDAHFD-fed mice. In addition, the phosphorylation of MST1/2 and YAP was significantly enhanced by EMPA treatment. Although there was no significant difference in the expression of YAP, we investigated whether a decrease in YAP phosphorylation results in a decrease in the expression of a transcriptional gene target. We observed a significant decrease in the mRNA expression of *Cyr61*, *cTGF*, and *AREG*. These findings indicate that EMPA activates the Hippo signalling pathway.

### 3.4. Empagliflozin Induced YAP Phosphorylation through the Hippo Signalling Pathway in LX-2 Cells

**Figure 4:**
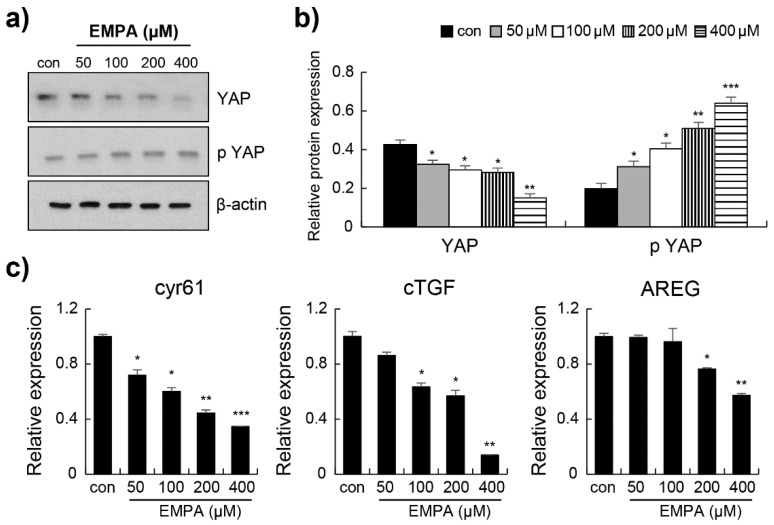
Empagliflozin decreased Yes-associated protein (YAP) expression in LX-2 cells. (**a**) The representative protein expression of YAP and phospho-YAP was determined using Western blot. (**b**) Quantitation and normalisation of phospho-YAP to total YAP or total YAP to β-actin from a. (**c**) The relative expression of *cyr61*, *cTGF*, and *AREG* was determined via RT-PCR. Data are expressed as means ± standard deviation of three independent experiments. * *p* < 0.05, ** *p* < 0.01, and *** *p* < 0.001 compared to the control.

**Figure 5:**
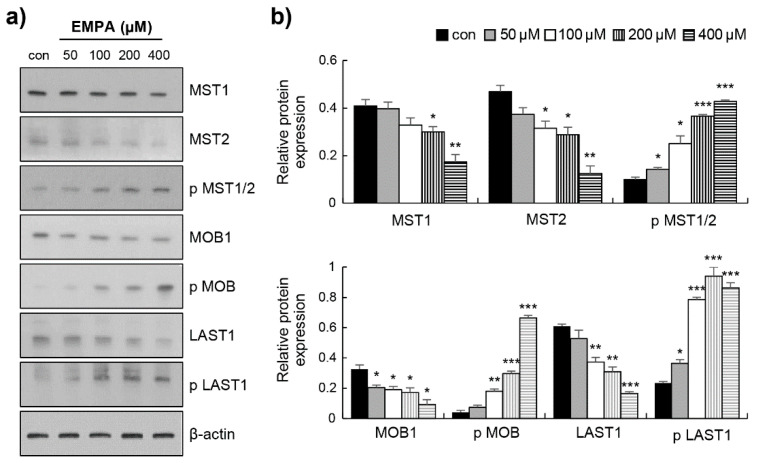
Empagliflozin regulates Hippo signalling in LX-2 cells. (**a**) Following different doses of EMPA treatment in LX-2 cells for 12 h, the protein levels of MST1, MST2, phospho-MST1/2, MOB1, phospho-MOB1, LATS1, and phospho-LATS1 were evaluated via Western blot. (**b**) Statistical analysis on the relative expression of the Hippo pathway in LX-2 cells. Data are expressed as means ± standard deviation of three independent experiments. * *p* < 0.05, ** *p* < 0.01, and *** *p* < 0.001 compared to the control.

To confirm the anti-fibrotic mechanism of EMPA observed in vitro, we investigated whether EMPA prevents fibrosis by activating the Hippo signalling pathway. First, Western blotting was performed to determine the YAP protein levels in LX-2 cells. EMPA induced a significant decrease in the total YAP protein level and an increase in the phosphorylation of YAP (Figure 4a,b). We also investigated the expression of its transcriptional gene targets: *Cyr61, cTGF,* and *AREG*. The mRNA expression of *Cyr61*, *cTGF*, and *AREG* was significantly decreased (Figure 4c). Next, the effect of EMPA treatment on the Hippo signalling pathway was investigated. As shown in Figure 5a,b, the levels of MST1, MST2, MOB1, and LATS1 were markedly reduced in EMPA treatment. Compared to those in the control group, EMPA treatment substantially enhanced the levels of p-MST1/2, p-MOB1, p-LATS1 (Figure 5). These findings indicate that EMPA can activate the Hippo pathway in stellate cells.

## 4. Discussion

In the present study, we demonstrated the effects of EMPA, an SGTL2 inhibitor, on hepatic fibrosis. Using hepatic stellate cells and a CDAHFD-induced liver fibrosis model, we provide evidence that EMPA alleviated liver fibrosis and inhibited hepatic stellate cell line proliferation and activation by activating Hippo signalling.

A key feature of the typical pathogenesis and progression of NAFLD is fat accumulation with inflammation and fibrosis [24]. A CDAHFD-induced mouse model has been used in many NAFLD and non-alcoholic steatohepatitis (NASH) studies [25,26,27]. A recent mechanistic study demonstrated that hepatocyte YAP deletion potently inhibits fibrosis development in carbon tetrachloride-induced liver injury in mice [28]. Moreover, in another study using a CDAHFD-induced NAFLD mouse model, hepatic CYR61 expression was increased in a YAP-dependent manner and was associated with fibrosis development [29]. In our previous experiments, we showed that EMPA ameliorated hepatic fibrosis in CDAHFD-induced NAFLD mice. Here, we investigated the role of the Hippo signalling pathway in the molecular mechanism of alleviating hepatic fibrosis using this mouse model.

SGLT2 inhibitors are a novel class of antidiabetic agents that inhibit renal tubular glucose reabsorption [30]. SGTL2 inhibitors protect against liver damage, inflammation, and steatosis based on experimental findings of patients with steatohepatitis and diabetes mellitus [31]. They also improve NAFLD and prevent disease progression [32]. EMPA has been suggested to be involved in the pathogenesis of NAFLD. EMPA reduces hepatic steatosis, which is associated with AMPK activation, as demonstrated in a T2DM animal model [33]. In addition, through the AMPK-TET2-autophagy pathway, EMPA reduces lipid accumulation and alleviates hepatic steatosis in human hepatic immortalised cells (HL-7702) and db/db mice [20]. We reported that EMPA decreased the secretion and expression of pro-inflammatory mediators via the IKK-NF-κB, MKK4/7-JNK, and JAK2-STAT1/3 pathways in LPS-induced RAW 264.7 macrophages [21]. We previously confirmed that EMPA treatment ameliorates hepatic steatosis, inflammation, oxidative stress, and fibrosis in CDAHFD-induced mouse models [22]. We then investigated the mechanism by which EMPA inhibits hepatic fibrosis.

The canonical Hippo signalling pathway is a highly conserved multi-protein kinase cascade associated with the MST1/2-SAV1 complex that phosphorylates and activates LATS1/2-MOB1A/B [7]. The activated LATS1/2-MOB1A/B complex phosphorylates and inactivates YAP/TAZ in mammals. In its phosphorylated form, cytoplasmic YAP is sequestered in an inactive state [8,10,11,12,13,14]. When the Hippo cascade is inactivated, YAP/TAZ acts as a transcriptional coactivator in the nucleus and enhances the transcription of genes, such as *Cyr61*, *cTGF*, and *AREG* [15]. According to a study, TAZ promotes the progression of NASH, including fibrosis, but without any difference in steatosis [34]. Zhang et al. reported that the expression of YAP is higher than TAZ in LX-2 cell lines [5]. Recently, a study reported that the hepatic expression of the YAP target genes was increased in NAFLD patients with fibrosis and CDAHFD-induced mice [29]. YAP is an important regulator for liver development and is an early and key regulator in the HSC activation process [16,35]. Recently, in addition to the canonical Hippo signalling pathway, MST1/2-LAST1/2-YAP/TAZ and NDR1/2 were identified as important members of the Hippo-YAP signalling pathway [9]. NDR1 promotes the inflammatory response by modulating the interleukin (IL)-17-mediated activation of NF-kB and interacts with TRAF3, which interferes with the IL-17R-Act1-TRAF6 complex [36]. The IL-17A-IL-17RA axis, which mediates the crosstalk between metabolically injured hepatic macrophages, hepatocytes, and fibrogenic myofibroblasts, plays a central role in alcohol-related liver disease [37]. Moreover, IL-17 plays a crucial role in the most frequent fibrotic liver disease [38].

In this study, we demonstrated that EMPA prevents hepatic fibrosis by activating the canonical Hippo-YAP pathway. Our results showed that EMPA greatly improved the state of liver fibrosis, as determined through macroscopic examination, H&E staining, Sirius Red staining, and immunohistochemical staining for α-SMA. EMPA decreased the expression of pro-fibrotic genes such as *α-SMA*, collagen1α1, *MMP2*, and *TGF-β*. Furthermore, EMPA successively induced the phosphorylation of MST1/2 and YAP, the two central members of the Hippo signalling pathway, in the CDAHFD-induced liver fibrosis model.

The key event in the development of liver fibrosis is the activation of HSCs [39]. HSC activation results in excessive ECM deposition and collagen production [40]. Therefore, one way to reduce fibrosis is to prevent the activation of HSCs. The phenotype of LX-2 cells is most similar to that of activated cells in vivo and expresses α-SMA and matrix metalloproteinase in all culture conditions [5]. We investigated the effects of EMPA on LX-2 cells. The results showed that EMPA significantly suppressed LX-2 viability in a dose-dependent manner. Furthermore, consistent with the results observed for CDAHFD-induced liver fibrosis, EMPA reduced the expression of pro-fibrogenic genes in LX-2 cells. Our results indicate that EMPA inhibits HSC activation, which is partly related to the regulation of proliferation, eventually reducing fibrogenesis. When we investigated whether EMPA inhibits the activation and proliferation of HSCs through Hippo signalling, EMPA was found to successively phosphorylate MST1/2, MOB1, LAST1, and YAP in LX-2 cells. Furthermore, EMPA significantly decreased the mRNA expression of *Cyr61*, *cTGF*, and *AREG*.

In conclusion, we discovered the anti-fibrotic effect of EMPA and established, for the first time, that the Hippo-YAP pathway is a crucial signalling pathway in vivo and in vitro. EMPA attenuates diet-induced hepatic fibrosis in an animal model and inhibits hepatic stellate cell proliferation and activation through the phosphorylation of downstream proteins via the Hippo signalling pathway. Overall, our results suggest a novel mechanism that could explain the anti-fibrotic action of EMPA.

## Data Availability

The data used to support the findings of this study are available from the corresponding authors upon reasonable request.

## Supplementary Materials

The following supporting information can be downloaded at: https://www.mdpi.com/article/10.3390/biomedicines10051032/s1, Figure S1: Empagliflozin exhibit no cytotoxic effect on LX-2; Table S1: Primer sequences used for quantitative real time-PCR.
