# Supplementary material for: Empagliflozin Reduces the Progression of Hepatic Fibrosis in a Mouse Model and Inhibits the Activation of Hepatic Stellate Cells via the Hippo Signalling Pathway
Source: Biomedicines. 2022 Apr 29;10(5):1032. doi: 10.3390/biomedicines10051032 (PMC9138578; doi:10.3390/biomedicines10051032)
Supplement: Supplementary file 1 [file biomedicines-10-01032-s001.zip › biomedicines-1679881-supplementary.pdf]

Table S1. Primer sequences used for quantitative real time-PCR

Gene	*Primer forward*	*Primer reverse*
Human		
α-SMA	TCCCTTGAGAAGAGTTACGAGTT	ATGATGCTGTTGTAGGTGGTT
Collagen1α1	AAT CCA TCG GTC ATG CTC TC	GGC CCA GAA GAA CTG GTA CA
TGF-β	GCAACAATTCCTGGCGTTACCT	GAAAGCCCTGTATTCCGTCTCC
Cyr61	AGCCTCGCATCCTATACAACC	TTCTTTCACAAGGCGGCACTC
cTGF	CCAATGACAACGCCTCCTG	TGGTGCAGCCAGAAAGCTC
AREG	TCCACTCGCTCTTCCAACAC	GGCATTTCACTCACAGGGGA
PCNA	GCGTGAACCTCACCAGTATGT	TCTTCGGCCCTTAGTGTAATGAT
Mouse		
α-SMA	CTGACAGAGGCACCACTGAA	CATCTCCAGAGTCCAGCACA
Collagen1α1	GAGCGGAGAGTACTGGATCG	GCTTCTTTTCCTTGGGGTTC
TGF-β	TTGCTTCAGCTCCACAGAGA	TGGTTGTAGAGGGCAAGGAC
MMP2	AGACACTGGTCGCAGTGATG	AGCTCCTGGATCCCCTTGAT
Cyr61	GACTGCAGCAAAACTCAGCC	CCAAGACGTGGTCTGAACGA
cTGF	GGCAACCCACTGATCCATCT	GGGCAGAAAGTTGGTGTCCT
AREG	GAATCGCTTTCTGGGGACCA	ATAGCTGCGAGGATGATGGC

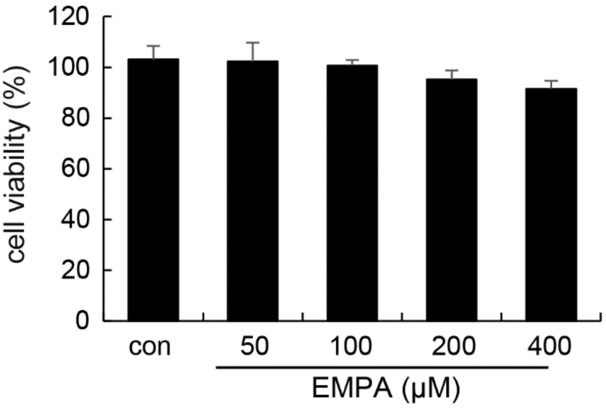

Figure S1. Empagliflozin exhibits no cytotoxic effect on LX-2. LX-2 cells were assessed for cytotoxic effects via the CCK-8 assay. LX-2 cells grown in a culture medium were treated with 50-400 μM of EMPA, respectively, for 24 h. Data are presented as means ± SD for the triplicate experiments.
